# PON-tstab: Protein Variant Stability Predictor. Importance of Training Data Quality

**DOI:** 10.3390/ijms19041009

**Published:** 2018-03-28

**Authors:** Yang Yang, Siddhaling Urolagin, Abhishek Niroula, Xuesong Ding, Bairong Shen, Mauno Vihinen

**Affiliations:** 1 School of Computer Science and Technology, Soochow University, No. 1. Shizi Street, Suzhou 215006, China; yyang@suda.edu.cn (Y.Y.); 20154227019@stu.suda.edu.cn (X.D.); 2 Department of Experimental Medical Science, BMC B13, Lund University, SE-22 184 Lund, Sweden; siddhaling@dubai.bits-pilani.ac.in (S.U.); abhishek.niroula@med.lu.se (A.N.); 3 Center for Systems Biology, Soochow University, No. 1. Shizi Street, Suzhou 215006, China; bairong.shen@suda.edu.cn

**Keywords:** protein stability prediction, variation interpretation, mutation, benchmark quality, machine learning method

## Abstract

Several methods have been developed to predict effects of amino acid substitutions on protein stability. Benchmark datasets are essential for method training and testing and have numerous requirements including that the data is representative for the investigated phenomenon. Available machine learning algorithms for variant stability have all been trained with ProTherm data. We noticed a number of issues with the contents, quality and relevance of the database. There were errors, but also features that had not been clearly communicated. Consequently, all machine learning variant stability predictors have been trained on biased and incorrect data. We obtained a corrected dataset and trained a random forests-based tool, PON-tstab, applicable to variants in any organism. Our results highlight the importance of the benchmark quality, suitability and appropriateness. Predictions are provided for three categories: stability decreasing, increasing and those not affecting stability.

## 1. Introduction

Stability of biomolecules, especially of proteins, is of great interest and significance. Stability in cells is important for the function of proteins in ambient conditions, responses in signalling and metabolic networks, as well as for many other features. Stability has been the major target for protein engineering, mainly to increase thermal stability [1,2], but sometimes also to destabilize proteins [3,4,5]. Effects on stability have been estimated to be among the most common consequences for disease-related variations [6], thus, stability prediction is of interest for variation interpretation to explain the effects of harmful variants.

Amino acid substitutions (AASs) affect biological, chemical and physical properties of proteins, including stability. Understanding the effects of these alterations facilitates the elucidation of molecular bases of many diseases. Site-directed mutagenesis has been utilized for decades to study and modify thermostability; however, the experimental trial and error-based design and construction of variants is time-consuming and expensive. Experimental methods are tedious and often costly, so there is need for computational methods that can predict effects of large numbers of AASs.

Several methods are available for predicting protein thermal stability changes due to AASs based on the primary sequence or protein three-dimensional structure information or both of them. Energy function-based methods include those using physical energy function from ab initio quantum mechanics (QM) calculations, empirical energy function or force field methods, or statistical energy function methods. The other major category of methods is machine learning (ML)-based methods. These tools either predict the sign of ΔΔG, i.e., whether AASs are stabilizing or destabilizing, or they calculate with regression the ΔΔG, or both. ML methods have been developed by using various algorithms including support vector machines [7,8,9,10], neural networks [11,12], gradient boosting [13], random forests (RF) [14,15] and a metapredictor [16]. Common to all these tools is that they have been trained with data from the same source, ProTherm database [17]. ProTherm contains detailed information for variants that come from numerous investigations.

The performance of ML methods is largely dependent on the quality of the data used for training and testing [18,19]. Benchmark datasets should fulfil a number of requirements [20]. The datasets should be relevant, representative, non-redundant, contain experimentally determined cases both for positive and negative effect, be scalable and allow reusability. A number of subselections of ProTherm have been used for method training and testing and are available at VariBench database [20], the dedicated resource for variation datasets.

Recently, it became evident to us that the ProTherm data contains a number of problems, which can hamper reliable predictor development. Some authors have applied certain measures to clean the data e.g., by selecting representative cases or by calculating averages over related entries [7,8,11]. However, systematic analysis of the relevance and quality of the dataset has been missing. Another area related to stability predictors and where improvements would be needed has been the lack of comprehensive reporting of the method performance [21,22] which has made it impossible to compare method performance reported in literature. Systematic performance analyses of several stability predictors [23,24] showed the tools to have widely varying performance. Even the best methods had only moderate accuracies.

Here, we checked thoroughly variant details in ProTherm database and corrected numerous problems. In the end, we had 38% of the original number of variants left. Out of these, 77% came from ProTherm, the rest are either corrected or new variants. Using the new high-quality dataset, we trained a novel ML-predictor, PON-tstab, for amino acid substitution effects on stability and established a new baseline for variant stability prediction method performance. Our study revealed the importance of knowing and checking data and their relevance when used for predictor development since predictors cannot be better than the data used to train them. Therefore, one has to be careful when using datasets collected by others unless they are properly documented and systematically compiled.

## 2. Results

### 2.1. Cleaning and Pruning Stability Data

**Figure 1:**
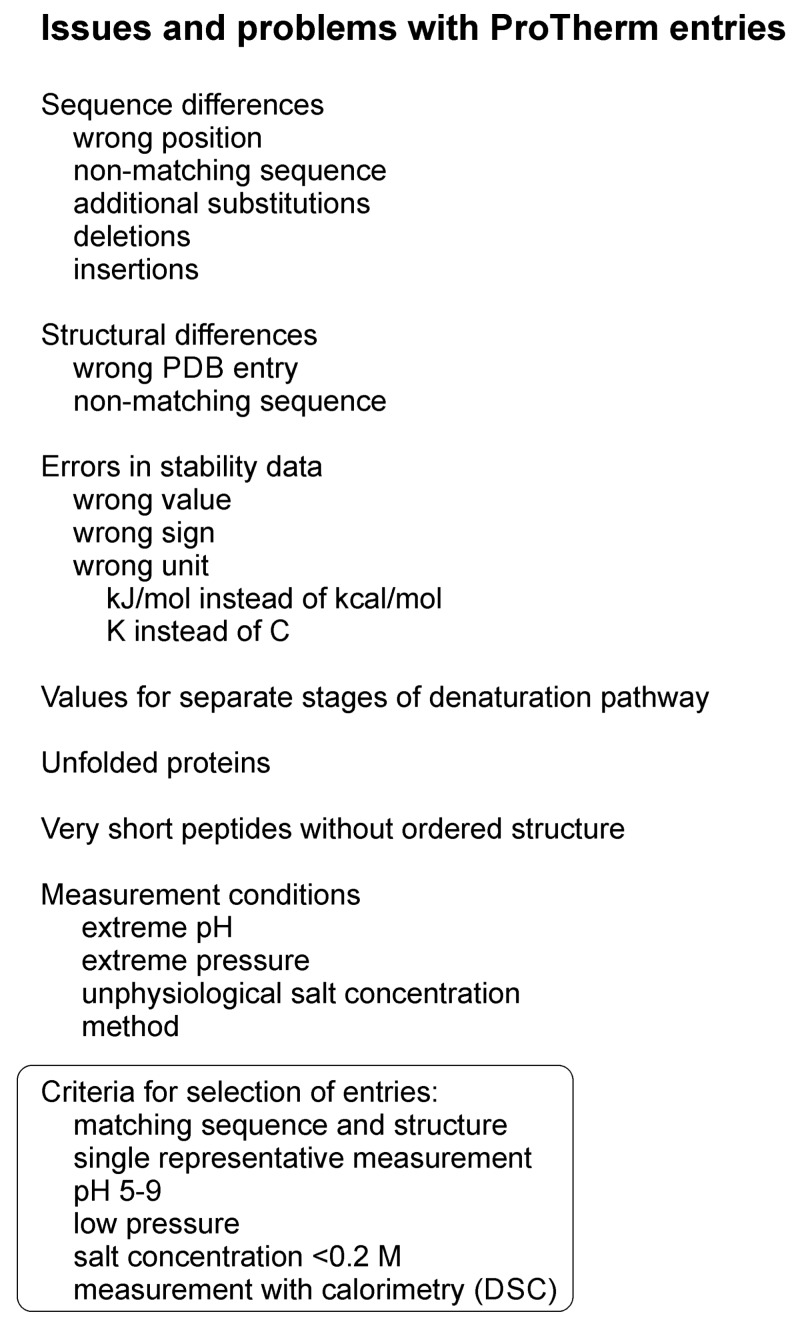
Types of problems and issues noted in ProTherm and which were taken into account when selecting an unbiased dataset for method training and testing. PDB, Protein Data Bank.

All the existing machine learning (ML) methods for protein variant stability prediction have been trained with data from a single database, ProTherm. We had previously noticed some issues with the database and wanted to correct them. While doing that, we noted additional issues and ended up in checking most of the entries having measured ΔΔG values and comparing them to the original literature. We found some errors, while some aspects are apparently features of the database; however, not clearly described and therefore the data has been used in wrong way. As an example of a feature can be mentioned that there are cases where for a two-stage denaturation pathway has been recorded values for the steps from folded to intermediate/transition stage, from intermediate to unfolded, and then the total value for the two. Previously, method developers have either taken the middlemost value or calculated an average over all of them, both practices being wrong. Examples of this kind of cases include variants in α-subunit of tryptophan synthase [25,26] and in apoflavodoxin [27]. The types of problems and issues noticed in the ProTherm entries are listed in Figure 1.

We found a number of instances where the sequence did not match with the one given in ProTherm, because there were additional variations. The most common problems of this kind include deletions, especially in the protein N-terminus. For example, from the murine interleukin-6 totally 22 N-terminal residues were removed [28]. Some proteins contain insertions, such as C-terminal FLAG-affinity tag in *Staphylococcal* nuclease, *Escherichia coli* ribose-binding protein and in *E. coli* maltose-binding protein [29], or additional residues such as in the bovine ribonuclease A [30].

In the case of T4 lysozyme, for which hundreds of variants together with three dimensional structures have been determined, the variants have been made to one of two backgrounds. The WT is the normal wildtype, whereas pseudo-wild type (WT*) is cysteine free and contains additional variants C54T and C97A [31] that remove all cysteines from the protein. For many variants, a wrong structural reference was given. Since many of the prediction methods use information about the sequence and variation context, it is essential to have the correct sequence and structure.

In some experiments, unfolded proteins were investigated such as subtilisin BPN’ variants at pH 5 [32]. These were discarded due to not representing a real folded protein. Still they have been included into several selections used for method development.

Several stability values had wrong sign or wrong unit. In ProTherm, a positive ΔΔG value indicates that the variant is stabilizing whereas a negative ΔΔG indicates the destabilizing effect of the variant. The majority of the measurements were indicated in kcal/mol, but there were numerous instances in kJ/mol, which have been previously used without conversion. Some measurement temperatures were given in Kelvin instead of Celsius. All these were corrected and unified.

Numerous entries were corrected, and others deleted. A number of entries for very short peptides were removed, as they do not represent true proteins with a defined fold. We generated rules for the selection of representative and reliable set of cases (Figure 1). We chose cases that were measured preferably with thermal denaturation methods, especially differential scanning calorimetry (DSC). Measurements performed in non-natural pH, high salt concentration or in high pressure were deleted if values for measurements in natural environment were available. The intacellular pH is controlled even in extremophilic micro-organisms, therefore the preferred pH range was 5–9, and salt concentration <0.2 M. We favoured single variants in comparison to background. The reference sequence had to match with the used sequence. In case the background sequence contained several variations common for several measured cases, we made the relevant changes to the reference sequence. Similarly, the three-dimensional structure had to match with the actual sequence. The dataset entries were checked and cleaned of duplicates.

The appearance of the large number of inconsistencies in ProTherm means that all previous machine learning methods have been trained with somewhat biased and incorrect data, although some cleaning and pruning may have been applied. Therefore, our cleaned dataset provides the baseline for determining the “new normal” of the stability predictor performance.

Previously several sub-selections of ProTherm data have been generated (see Supplementary Material), available at the VariBench database. Most of the previous selections have been for single variants in proteins. However, none of them reports correcting for additional variants, as we did. Some of these selections have been corrected for errors in units, but not all. The pH range has been limited close to neutrality in several datasets, and some have restricted the temperature range, which may have biased dataset towards proteins functional at rather low temperatures. Most common way of dealing with several measurements for the same variants has been taking an average of them. The second most common selection has been for representative cases. Many datasets have contained duplicates.

Structural information has been a requirement for a number of datasets, however, e.g., the background (WT vs. WT*) has been noted in only one. And even in that case, apparently no corrections were made. We found several cases where the reference sequence and structure were wrong. Some datasets have excluded variants with large ΔΔG values, melting temperature differences or long sequences.

None of the previous selections have utilized as extensive cleaning and pruning approach as we used. The following selection criteria have not been applied previously to any of the datasets: wrong sequence position, non-matching sequence, additional sequence changes including deletions and insertions, wrong Protein Data Bank (PDB) reference, non-matching PDB structure, values for separate stages of denaturation pathway, unfolded proteins, and very short peptides (see Figure 1). Thus, our selection is much more extensive and detailed and therefore representative of true data, meaning that the new dataset is best suited for testing and training prediction methods.

### 2.2. Dataset Properties

The final dataset contains 1564 entries from 99 proteins. This dataset is available in VariBench at http://structure.bmc.lu.se/VariBench/stability.php [20]. The proteins in the dataset originate from a wide spectrum of organisms. There are 233 stabilizing and 864 destabilizing variants, while 467 do not affect stability. Because of technical difficulties in measuring exact ΔΔG values [33], we consider cases with values between −0.5 and 0.5 kcal/mol as neutral, similar to our previous studies [7,23].

The largest numbers of variants are for lysozyme (250), barnase (132), gene V protein (111), cold chock protein (101) and chymotrypsin inhibitor (100). The ΔΔG values range from −17.4 to 23.0 kcal/mol, measurement pHs from 2.7 to 9.6, the majority being between 5 and 8. The measurement temperatures range from 0 to 89 °C. Thus, the included cases represent a wide spectrum of proteins and measurement conditions and thus provide a good starting point for method development.

Of the 4148 entries in the ProTherm database with values for ΔΔG, 1197 (29%) are included as such into our selection. In addition, we have 367 entries that either had errors in ProTherm or were missing. Altogether, 2951 entries were not approved to the final dataset due to the problems discussed above or because of being duplicates or otherwise not fulfilling the inclusion criteria. The new dataset is smaller than the most ones used previously for method training; however, it is consistent and has been cleaned of several problematic cases and mistakes, and as such provides an unbiased starting point for method development.

The amino acid distribution of the variants in the dataset is shown in Supplementary Table S1. Alanine scanning mutagenesis has been widely used for investigating the function of amino acids. These variants are clearly the most common in our dataset, altogether 410 instances (26%) spread out over all possible original residues. The other amino acid types for substituting residues are quite equally represented in the dataset. Glycine (120), valine (113) and serine (80) are the next most common substituting amino acids.

The dataset has its limitations and so does the entire ProTherm. There are a number of substitutions that are not represented at all. These follow to some extent the genetic code, only 150 of the total of 380 amino acid substitutions are possible by single nucleotide substitution in DNA. Valine (175), aspartate (135), glutamate (124), and leucine (115) are the most frequent substituted residues. Substitutions from tryptophan (18), cysteine (32), glutamine (36) and methionine (37) are the least frequent ones.

### 2.3. Novel Stability Predictor

**Figure 2:**
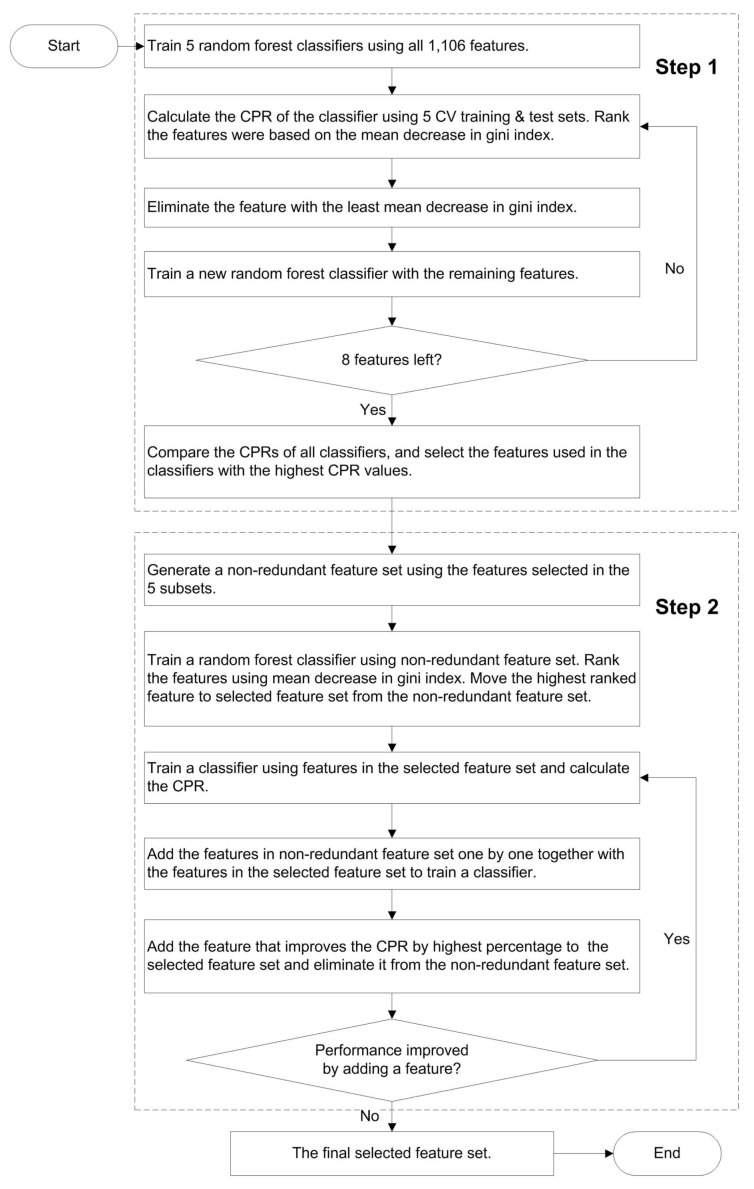
The procedure for feature selection. CPR, correct prediction ratio; CV, cross validation.

In total, 1106 features were collected to train a machine learning method for stability prediction. To eliminate redundant and non-relevant features, we used a combined greedy feature-selection algorithm with two steps (Figure 2): backward elimination and forward selection. This approach has been previously described [34].

The data were partitioned to subclasses as follows. First an independent test set of 165 variants in ten proteins was separated. The proteins in this dataset did not have close homologues (>30% sequence identity) with each other or rest of the proteins. The remaining cases were used for training (1399 variations in 89 proteins). For that purpose, the data were split into five partitions of approximately equal sizes and used in five-fold cross validation (Supplementary Table S2). Variations from the same protein and closely related proteins were always kept in the same partition during the five-fold cross validation. In each cross-validation step, four partitions were used for training and the remaining partition for testing.

In the first feature selection step, 5 feature subsets were independently selected by using the cross-validation training and test sets (Figure 2). Each feature selection consisted of a loop where a RF classifier was used to eliminate one feature per iteration. This was repeated until 8 features were left. The number was chosen based on our prior experience of highly reliable predictor development [7,34,35,36,37]. In all these methods, the number of informative features has been ten or less. Then the features that gave the best performance were selected. All the selected features were merged, and duplicates were eliminated. In the second feature selection, a single feature subset was selected by using all the five cross-validation training and test dataset partitions. The most useful feature was selected by training a classifier and tested using the cross-validation datasets. In the next step, each of the remaining features was combined with the selected feature(s) one at a time and the feature that showed the least error rate was selected. This was repeated until the addition of features no more reduced the error rate from the previous iteration. After that, the selected features were used for training the final predictor.

### 2.4. Predictor Training

When using all the features for three-class prediction (stability increasing, decreasing and not affecting stability), the ratio of correctly predicted cases is 0.466 and 0.469 after normalization in 5-fold cross validation, and increases to 0.573 after feature selection (Table 1). Note that a random prediction for three states would have a score of 0.33 for equally distributed data. As this was not the case with our dataset, we normalized the categories to be of equal size. This predictor was based only on eight features, including temperature, one similarity feature, three amino acid features, two-neighbour features and one protein feature (Supplementary Table S3). As the numbers of cases in the categories are different, we had to normalize them to get comparable results. After normalization, the correct prediction ratio (CPR) was decreased to 0.450 (Table 1). We used the no-change category as the reference state based on which the stability increasing and decreasing cases were normalized.

**Figure 3:**
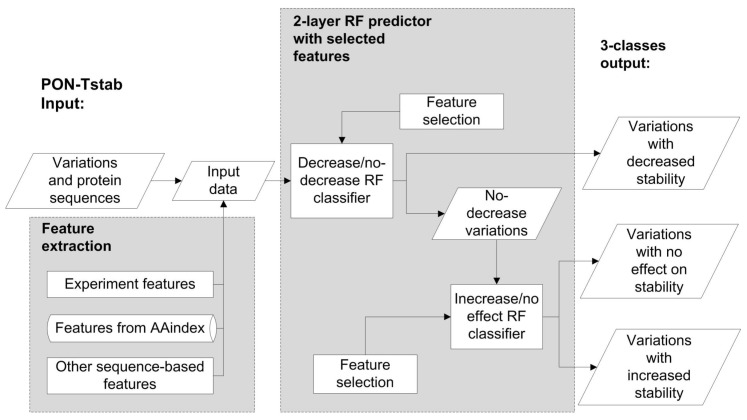
The scheme for the PON-tstab predictor. A two-layer random forest predictor was developed to predict increasing, decreasing or having no change on variant stability. RF, random forest.

To further improve the method, we generated two binary classifiers based on a balanced dataset which has been shown to be beneficial even when the true classes have different frequencies [38]. For the first classifier we combined the same number of variations in classes increase and no effect on protein stability together as no decrease, the total number of no increase variations was the same as that of decrease cases. The second classifier was trained on balanced increase and no effect cases. Then we performed feature-selection separately for both of them. The accuracies for decrease/no decrease and increase/no effect classifiers are 0.67 and 0.62, respectively. 8 Features were informative for the decrease/no decrease classifier while 3 features were informative for increase/no effect classifier. The feature temperature is included to both classifiers, so altogether there are 10 unique features (Supplementary Table S3) that were used to generate the 2-layer predictor (Figure 3). First, the tool predicts whether a variant leads to decreased stability or not. Variants that do not decrease stability are then classified further into those that increase or have no effect on stability. We call this method for PON-tstab.

Reverse variants have been suggested to improve prediction performance [39]. We trained four predictors including reverse variants, however the performance was consistently somewhat lower than for the original variants only (Table 1). This may be due to altered variant context in the new reference sequences.

### 2.5. Testing

For the two-layer predictor without feature selection, the normalized CPR and GC^2^ are 0.459 and 0.085, respectively, and increase to 0.503 and 0.112 after feature selection by five-fold cross validation. The two-layer predictor with ten features (PON-tstab) had the best performance.

We further tested the method with the blind test set that was selected in the beginning and not used in any of the previous steps. The dataset contains 165 variations. PON-tstab has better performance than the three other versions (Table 2). After normalization, CPR and GC^2^ are 0.429 and 0.219, respectively, for PON-tstab.

We compared the performance of PON-tstab to that for some previously published tools. These include I-mutant 2.0 [9], INPS [10] and EASE-MM [8]. They all have been trained with datasets extracted from ProtTherm and thus are based on several problematic and erroneous cases.

We excluded from the blind test dataset of 165 variant records those that had been used for training the other predictors. In the end, we had 40 variants that came from 5 proteins and did not have even close homologues among our training dataset proteins. The performance of the 4 predictors is shown in Table 3. Both EASE-MM and I-Mutant 2.0 have CPR of 0.6 before normalization, whereas the GC^2^ is very low for both the tools. After normalization, the CPR is much lower, 0.36 for EASE-MM and 0.37 for I-Mutant 2.0 and 0.43 for PON-tstab. INPS could predict only 15 out of the 40 variants, 9 of which were correctly predicted. Since none of these variations increase protein stability, the GC^2^ score cannot be calculated. Therefore, the result cannot be normalized, either.

## 3. Discussion

ML methods are trained on known cases, thus, these instances should be reliably defined. Datasets for training, testing and benchmarking have a number of requirements, as previously described [20], here the relevance was investigated. During the analysis of cases in ProTherm database it became apparent that it was necessary to perform an exhaustive check to select correct and representative cases.

Some of the noticed issues are features of the database; however, either not known or taken into account by method developers. Problems with data selection have been further inherited to other studies when data have been used without controlling the quality. There are numerous such examples in the protein stability prediction field. All the existing selections turned out to contain numerous problematic cases. Thus, available ML predictors have been trained on non-optimal data and therefore their performance is adversely affected. Further, the performance tests have been biased and results inflated.

We corrected numerous problems and included additional variants to obtain a consistent and reliable set. There are 1563 variants, only about half of what has been used to train some recent methods. With this new dataset we can obtain reliable estimates of method performance, as well.

The new dataset has uneven distribution of substitution types. The same problem appears in all the previous ProTherm selections, as well. Since some substitutions are not represented at all and others just by a few cases, it is apparent that prediction methods have limitations. New variant stability cases are published infrequently. We can expect the spread of massively parallel reporter assays (MPRAs) to improve the situation in the future. Much larger datasets would be needed to cover the context effects in protein sequences and three-dimensional structures. At the moment the landscape is very sparse.

The performance measures for PON-tstab are lower than those recently reported for some predictors. However, as only PON-tstab has been trained on quality controlled and unbiased data, our results have to be considered as the current state-of-the-art for variant stability predictors. The performance is somewhat skewed. NPV values are clearly better than PPV values. There are wide differences on the performance measures for increasing, decreasing and not affecting variants. It is evident that this is due to the size and composition limits of the dataset. Although the ProTherm database has not been updated for five years, it is still up-to-date because stability details are seldom published.

There are likely several reasons why the performance of the new method seems to be lower than what has been claimed for some previous methods. Use of biased datasets may provide benefits, especially as some previous method developers have used circulatory cases which increase the performance. Some tools have included variants in the same position in the training and test data. As many features are protein wide they apply to all variants in a position. We had most stringent test, the proteins with variants did not even have close homologs among training data. Probably one of the major factors limiting the performance of stability predictors is the size of the dataset. Our selected dataset includes just 1564 variants. Protein stability is a complex property and numerous factors contribute to it. Natural proteins have numerous stabilizing bonds and interactions, still the free energy difference between the folded and unfolded form is only 3 to 15 kcal/mol [40]. To fully capture the features governing the stability would require substantially larger dataset.

Developers of ML methods have to know their data and trust on it. As this example indicates, the quality of data has to be validated. This is especially important when using datasets developed by others.

When training PON-tstab, lessons from our previous machine learning methods [7,34,35,36,37,41] were taken into account. We are confident that it represents the highest performance attainable with the currently available data. Only with a substantial increase in learning and test dataset sizes, we can expect to improve the performance. We used the largest number of features ever applied to stability predictors; however, just ten features are informative. Despite its limitations, PON-tstab is useful for estimating variant effects whether for interpreting consequences of disease-related substitutions or planning for stability changes to modify protein properties.

## 4. Materials and Methods

### 4.1. Variation Data

Stability affecting variants were taken from ProTherm database (the latest update was from 22 February 2013). Both manual and computational steps were applied to check the correctness and quality of annotations. We corrected inconsistencies with data in published articles and included additional cases. The sequence for the investigated protein was matched with sequence entries in UniProtKB [42]. The protein three dimensional structures were chosen to have the background sequence used in experiments. Sometimes additional variants appeared in sequences in comparison to the wild type form of the protein.

A selection of reliable cases was made. If there were several entries for a variant, the criteria for selecting cases included the following: experimental pH close to neutrality, low salt concentration, stability determined with thermal denaturation method, preferably with a calorimeter (Figure 1). The final set was manually curated and used to develop a novel stability predictor.

### 4.2. Features

The collected featured to characterize the variants can be classified as follows:

The first two features are for experimental conditions: temperature and pH value.

Conservation features. Three features were from information content and position specific scoring matrices. First, we collected related sequences by a Basic Local Alignment Search Tool (BLAST) [43] search against the non-redundant protein sequence database at National Center for Biotechnology Information (NCBI). The significant sequence hits (E-value < 0.001) were aligned using ClustalW [44]. Based on the multiple sequence alignment (MSA) of the protein sequences, position specific scoring matrix (PSSM) and information content for each position of the reference protein sequence were generated using the AlignInfo module in BioPython. The information content and PSSM scores for the reference and the altered amino acid at the variant site were used as features [36,37]. These features were previously described in [36,37]. Another 4 co-evolution features were calculated by CAPS tool [45] using the protein sequences obtained from the BLAST search. These features (is_coevolving, max_cor, is_coevolving_grp, grp_count) indicate if the original residue at the variant position has co-evolved with any other residue in the protein, the correlation coefficient of co-evolution, if the residue is part of any of co-evolving groups found by CAPS and the number of these groups, respectively.

Amino acid features. The AAindex database contains totally 685 features in 3 databases, 544, 94 and 47 features, respectively [46]. AAindex contains indices for physicochemical properties and propensities of amino acids, substitution matrices and pair-wise contact potentials. Incomplete feature sets were eliminated, leaving 617 features, similar to those used for training the PON-P2 predictor [34].

Variation type features. Two matrices were used for the types of the amino acid substitutions. The first one is a 20 × 20 matrix with the first dimension denoting to original residue and the other to 20 amino acids for variant residue. In this matrix, only one position has value 1 while all others are set to 0. Another matrix has 6 × 6 size. In this case, the amino acids are divided into 6 groups according to their physicochemical properties, as follows: hydrophobic (V, I, L, F, M, W, Y, C), negatively charged (D, E), positively charged (R, K, H), conformational (G, P), polar (N, Q, S) and others (A, T) [47]. Similar to the previous set of features, the feature corresponding to the original and variant residues is set to 1 and the remaining features are set to 0.

Neighbourhood features. To take into account the sequence context of variation sites, 25 features were included. 20-dimensional vector of neighbourhood residues indicates the occurrences of each amino acid type within the neighbourhood in a window of 23 positions, i.e., 11 positions both before and after the variation site.

Five additional neighbourhood features included NonPolarAA, PolarAA, ChargedAA, PosAA and NegAA, which denote to the numbers of nonpolar, polar, charged, positively charged and negatively charged neighborhood residues [48] within a window of 23 residues, respectively.

Other sequence-based protein features. 19 features for either thermodynamic indices of the extended protein state or property-based indices obtained with ProtDCal protein descriptor calculator [49] are listed in Supplementary Table S4.

Altogether 1106 features were collected and a two-step feature selection was performed to find the useful features for stability prediction.

### 4.3. Predictor Training

Random forest (RF) algorithm implemented in the R package [50] was used for classification and regression. RF combines tree predictors such that each tree depends on the values of a random vector sampled independently and with the same distribution for all trees in the forest. RF can be applied to multi-class classification tasks as well as to regression.

The default number of trees grown in each random forest was set to 300 and the default values were used for all other parameters. These settings have been discussed in our previous work [41]. 

The training and test datasets are available at VariBench database for variation datasets [20] at http://structure.bmc.lu.se/VariBench/stability.php.

### 4.4. Method Quality Assessment

For comprehensive reporting of the predictor, we used a number of measures as previously suggested [22,51], including

(1) $$Accuracy=\frac{\mathrm{TP}+\mathrm{TN}}{\mathrm{TP}+\mathrm{TN}+\mathrm{FP}+\mathrm{FN}}$$

Positive predictive value

(2) $$PPV=\frac{\mathrm{TP}}{\mathrm{TP}+\mathrm{FP}}$$

Negative predictive value

(3) $$NPV=\frac{\mathrm{TN}}{\mathrm{TN}+\mathrm{FN}}$$

Sensitivity/True positive rate

(4) $$TPR=\frac{\mathrm{TP}}{\mathrm{TP}+\mathrm{FN}}$$

Specificity/True negative rate

(5) $$TNR=\frac{\mathrm{TN}}{\mathrm{TN}+\mathrm{FP}}$$

and Matthews Correlation Coefficient:

(6) $$MCC=\frac{\left(\mathrm{TP}\times \mathrm{TN})-(\mathrm{FP}\times \mathrm{FN}\right)}{\sqrt{\left(\mathrm{TP}+\mathrm{FN})\times (\mathrm{TP}+\mathrm{FP})\times (\mathrm{TN}+\mathrm{FN})\times (\mathrm{TN}+\mathrm{FP}\right)}}$$

where TP, TN, FP, and FN represent the numbers of true positives, true negatives, false positives, and false negatives, respectively. Matthews correlation coefficient (MCC) was used to evaluate the performance of the two binary classifiers in PON-tstab.

For 3-class predictors, we used correct prediction ratio (CPR) instead of accuracy. The generalized squared correlation, GC^2^, was used since MCC is not readily generalized to more than two classes [52]. The squared correlation is calculated as

(7) $${GC}^{2}=\frac{\sum_{ij}\frac{{\left(z_{ij}-e_{ij}\right)}^{2}}{e_{ij}}}{N(K-1)}$$

where *z_ij_* represents the number of times the input is predicted to be in class *j* while belonging in reality to class *i*, and *e_ij_* = *x_i_y_j_*/*N* is the expected number of data in cell *i*,*j* is the expected number of data in cell *i*,*j* of the contingency matrix under the null hypothesis assumption that there is no correlation between assignments and predictions. *K* denotes to number of classes. *N* means number of total inputs while *x_i_* is the number of inputs predicted to be in class *i*. The values for GC^2^ range from 0 to 1 unlike MCC that ranges from −1 to 1.

Since the three classes in the test dataset were of different sizes (the proportion of increase, no effect and decrease samples is about 1:2:4, Supplementary Table S4), the numbers were normalized so that they equalled the size of class “no effect on stability”.

## Figures and Tables

**Table 1:** Comparison of different classifier designs on five-fold cross-validation.

Performance Measures		Predictors Trained and Test on Original Dataset				Feature Selection on Original Dataset and Training/Testing on Balanced Original and Reversed Dataset			
		3-Class RF with All Features ^a^	3-Class RF with 8 Selected Features	2-Layer Predictor with All Features	2-Layer Predictor with 10 Selected Features (PON-tstab)	3-Class RF with All Features	3-Class RF with 5 Selected Features	2-Layer Predictor on 3 Classifiers with All Features	2-Layer Predictor on 3 Classifiers with Selected Features
TP ^b^	+	21.6/43.5	9.6/19.0	19/38.3	23.2/46.3	40.8	44.8	42.8	43.4
	−	74.8/40.2	124.4/67.2	90.6/48.1	91.4/49.0	43.4	42	41.6	37.4
	no	34	26.4	28.8	30.8	36.2	37.2	37.8	40
TN	+	180.6/123.6	32.2/64.4	183.4/125.7	186.2/127.9	125.4	122.2	125.4	123.8
	−	95/130.4	30.2/16.2	85.8/118.1	88.2/122.7	125.4	129.2	126.4	131.4
	no	134.6/113.9	57	149/121.6	150.8/125.6	121.4	123.4	121.2	116.4
FP	+	57.4/43.2	10.6/8.0	54.6/41.1	51.8/38.9	41.8	45	41.8	43.4
	−	30.2/36.4	71.8/91.6	39.4/48.7	37/44.1	42.8	38	40.8	35.8
	no	61.8/52.9	37/38.0	47.4/45.2	45.6/41.2	45.8	43.8	46	50.8
FN	+	20.2/39.9	227.4/158.7	22.8/45.1	18.6/37.1	42.8	38.8	40.8	40.2
	−	79.8/43.2	53.4/75.2	64/35.3	63.2/34.4	40.2	41.6	42	46.2
	no	49.4	159.4/128.8	54.6	52.6	47.4	46.4	45.8	43.6
Sensitivity	+	0.516	0.228	0.455	0.554	0.488	0.537	0.511	0.518
	−	0.483	0.805	0.583	0.59	0.52	0.504	0.498	0.449
	no	0.406	0.318	0.339	0.367	0.432	0.445	0.451	0.478
Specificity	+	0.759/0.744	0.955/0.953	0.771/0.756	0.783/0.769	0.75	0.732	0.75	0.741
	−	0.755/0.776	0.427/0.451	0.68/0.700	0.701/0.730	0.743	0.772	0.755	0.785
	no	0.686/0.683	0.812/0.772	0.757/0.732	0.767/0.756	0.727	0.739	0.725	0.697
PPV	+	0.271/0.498	0.463/0.691	0.258/0.481	0.318/0.551	0.492	0.505	0.505	0.502
	−	0.714/0.527	0.635/0.424	0.701/0.503	0.715/0.530	0.505	0.528	0.548	0.513
	no	0.354/0.388	0.421/0.413	0.371/0.386	0.399/0.428	0.445	0.462	0.452	0.444
NPV	+	0.9/0.757	0.876/0.712	0.89/0.763	0.909/0.774	0.746	0.76	0.755	0.756
	−	0.543/0.75	0.643/0.824	0.57/0.771	0.58/0.781	0.756	0.758	0.75	0.741
	no	0.732/0.698	0.737/0.694	0.731/0.690	0.741/0.705	0.719	0.727	0.726	0.728
GC^2^		0.172/0.078	0.101/0.281	0.121/0.085	0.162/0.112	0.063	0.08	0.068	0.071
CPR		0.466/0.469	0.573/0.450	0.495/0.459	0.520/0.503	0.48	0.495	0.487	0.481

^a^ Normalized performance values are separated by a slash if they are different from the original ones. ^b^ GC^2^, generalized squared correction; CPR, correct prediction ratio; FN, false negative; FP, false positive; NPV, negative predictive value; PPV, positive predictive value; RF, random forest; TN, true negative; TP, true positive.

**Table 2:** Blind test performance.

Performance Measures		Predictors			
		3-Class RF with All 1106 Features ^a^	3-Class RF with 8 Selected Features	2-Layer Predictor with All 1106 Features	2-Layer Predictor with 10 Selected Features (PON-tstab)
TP	+	2/4.4	1/2.2	4/8.7	3/6.5
	−	66/35.9	69/37.5	62/33.7	66/35.9
	no	18	16	20	22
TN	+	135/94.8	134/94.7	120/83.5	126/88.1
	−	28/36.2	20/22.3	37/45.2	37/46.4
	no	88/77.2	97/88.6	94/83.7	93/79.9
FP	+	7/5.2	8/5.3	22/16.5	16/11.9
	−	45/63.8	53/77.7	36/54.8	36/53.6
	no	27/22.8	18/11.4	21/16.3	22/20.1
FN	+	21/45.7	22/47.8	19/41.3	20/43.5
	−	26/14.1	23/12.5	30/16.3	26/14.1
	no	32	34	30	28
Sensitivity	+	0.087	0.043	0.174	0.130
	−	0.717	0.750	0.674	0.717
	no	0.360	0.320	0.400	0.440
Specificity	+	0.951/0.948	0.944/0.947	0.845/0.835	0.887/0.881
	−	0.384/0.362	0.274/0.223	0.507/0.452	0.507/0.464
	no	0.765/0.772	0.843/0.886	0.817/0.837	0.809/0.799
PPV	+	0.222/0.457	0.111/0.292	0.154/0.345	0.158/0.354
	−	0.595/0.36	0.566/0.326	0.633/0.381	0.647/0.401
	no	0,4/0.441	0.471/0.584	0.488/0.551	0.5/0.522
NPV	+	0.865/0.675	0.859/0.665	0.863/0.669	0.863/0.670
	−	0.519/0.719	0.465/0.641	0.552/0.735	0.587/0.767
	no	0.733/0.707	0.74/0.723	0.758/0.736	0.769/0.740
GC^2^		0.049/0.291	0.091/0.476	0.043/0.200	0.046/0.219
CPR		0.521/0.388	0.521/0.371	0.521/0.416	0.552/0.429

^a^ Normalized performance values are separated by a slash if they are different from the original ones.

**Table 3:** Performance of PON-tstab and comparison to other methods.

		Predictors			
Performance Measures		EASE-MM	I-Mutant	INPS	PON-tstab
Variants Predicted		40	40	15	165
TP	+	0	0	0	3/6.5
	−	22/6.8	22/6.8	9	66/35.9
	no	2	2	0	22
TN	+	34/16	33/15.7	15	126/88.1
	−	2	3/3.3	0	37/46.4
	no	28/14.77	28/13.7	9	93/79.9
FP	+	0	1/0.3	0	16/11.9
	−	12/14	11/12.7	2	36/53.6
	no	4/1.23	4/2.3	4	22/20.1
FN	+	6/8	6/8	0	20/43.5
	−	4/1.2	4/1.2	4	26/14.1
	no	6	6	2	28
Sensitivity	+	0	0	NA ^a^	0.13
	−	0.85	0.85	0.69	0.717
	no	0.25	0.25	0	0.44
Specificity	+	1	0.97/0.98	1	0.89/0.88
	−	0.14/0.13	0.21	0	0.51/0.46
	no	0.88/0.92	0.88/0.86	0.69	0.81/0.80
PPV	+	0	0	NA	0.16/0.35
	−	0.65/0.33	0.67/0.35	0.82	0.65/0.40
	no	0.33/0.62	0.33/0.47	0	0.5/0.52
NPV	+	0.85/0.67	0.85/0.66	1	0.86/0.67
	−	0.33/0.62	0.43/0.73	0	0.59/0.77
	no	0.82/0.71	0.83/0.70	0.82	0.77/0.74
GC^2^		0.13/0.68	0.09/0.54	NA	0.05/0.22
CPR		0.6/0.36	0.6/0.37	0.6	0.55/0.43

^a^ Not available.

## Supplementary Materials

Supplementary materials can be found at http://www.mdpi.com/1422-0067/19/4/1009/s1.

## Abbreviations

ΔΔG	Gibb’s free energy
AAS	Amino acid substitution
BLAST	Basic Local Alignment Search Tool
CPR	Correct prediction ratio
DSC	Differential scanning calorimetry
FN	False negative
FP	False positive
GC^2^	Generalized squared correlation
MCC	Matthews correlation coefficient
ML	Machine learning
MPRA	Massively parallel reporter assay
MSA	Multiple sequence alignment
NPV	Negative predictive value
PPV	Positive predictive value
QM	Quantum mechanics
TN	True negative
TNR	True negative rate
TP	True positive
TPR	True positive rate
