# Supplementary material for: PON-tstab: Protein Variant Stability Predictor. Importance of Training Data Quality
Source: Int J Mol Sci. 2018 Mar 28;19(4):1009. doi: 10.3390/ijms19041009 (PMC5979465; doi:10.3390/ijms19041009)
Supplement: Supplementary file 1 [file ijms-19-01009-s001.pdf]

International Journal of *Molecular Sciences*

Supplementary Materials

Previous Protherm Selections

Many of the datasets have been used in several articles. Original papers reporting the datasets are described.

I-Mutant [1]

S1615 single AASs in 42 proteins with structures available.

S388 subset of the former, measurements at pH 6-8 and temperature 20-40°C. Variants in 17 proteins.

I-Mutant2.0 [2]

Single AASs with experimental measurements.

2087 with sequence information

1948 with 3D structures.

MUpro [3]

SR1135 Redundancy cleaned version of S1615 [1], removal of identical duplicates.

S388 Subset of S1615. Unique variants measured at physiological conditions.

SR1135 Subset of S1615. Removed parallel cases.

SR1023 Subset of S1615 from where identical variants were removed.

Saraboji *et al*. [4]

1791 single AASs. Secondary structure and solvent accessible surface available for PDB structure. Thermal denaturation method.

1396 variants with thermal denaturation and 2205 variants with chemical denaturation.

Experimental conditions (pH, ions, buffer, additives etc.) not considered.

iPTREE-STAB [5]

1859 single variants in 64 proteins. Duplicates were removed, and same variants in same conditions averaged.

SVM-WIN31 and SVM-3D12 [6]

1681 single AASs (sequences) in 58 proteins and 1634 in 55 proteins (structures available) both in reversible experiments.

499 additional variants from a later version of ProTherm, excluded new variants at the same positions as in the other datasets.

AUTOMUTE [7]

1204 and 1962 variants from 1396 and 2204 of [4] by removing cases which missed from PDB or had less than six nearest neighbours.

PoPMuSiC-2.0 [8]

2648 single AASs in 131 proteins. Original articles checked. Globular proteins, structure available. Only in true wild type background. Heme-containing proteins excluded (except apo forms), as well as variants involving prolines. Destabilizing variants with ΔΔG value larger than 5 kcal/mol excluded. Average value for parallel experiments. If the protein forms homo-multimers, values accepted only when monomer state verified. Measurements close to pH 7 and temperature close to 25°C, without additives, were favoured.

Potapov et al. [9]

2156 Single variants. Combined two datasets [10] and ProTherm. Removed from Guerois set cases not matching with PDB structure. The latter set filtered to exclude duplicates with the first set, to remove all structures determined with NMR. Parallel results for variants were averaged.

Khan and Vihinen [11]

1784 Single variants in 80 proteins. Representative cases selected for variants measured several times and in different conditions.

sMMGB [12]

1109 Variants in 60 proteins with 3D structures. Single AASs, measurement pH 6-8. Average value for parallel experiments.

M47 and M8 [13]

S2760 variants in 75 proteins. Single AASs with PDB structure available. Parallel results averaged.

S1810 variants in 71 proteins. Cases between -0.5 and 0.5 kcal/mol excluded from S2760.

EASE-MM [14]

1914 Single AASs in 95 proteins. Manually checked. Averaged values for variants measured in the same condition. Clustering of proteins.

S236 Subselection of I-Mutant2.0 dataset [6] including 25 proteins. Note that in the article the dataset is named as S238.

S1676 Remaining cases from the set of 1914.

S543 In 55 proteins [15]. Subset of 2648 [8]. <25% sequence identity to both S1676 and S236.

mCSM [16]

S350 Randomly picked subselection of S2648 [8]. Variants in 67 proteins.

S1925 Variants in 55 proteins. Uniformly distributed to SCOP classes.

42 Variants in P53 DNA binding domain with experimental details.

HoTMuSiC [17]

1626 Single AASs in 90 proteins. Manually checked.

Resolution of structure <2.5Å, ΔT_m measurements without chemical denaturants, proteins with two-state folding transition. Effects on stability larger than 20°C excluded.

More details at https://www.biorxiv.org/content/early/2016/01/10/036301

Jia et al. [18]

380 Single AASs. Measurements at pH 6-8. Proteins <300 AAs long. Multiple data points averaged. $\Delta\Delta G$ values <-10 or >10 kcal/mol excluded.

Numerous datasets for training and testing.

SAAFEC [19]

sDB, 1262 variants in 49 proteins. Measurements between pH 5-9, representatives for each variant. When several measurement values, those within 0.1 kcal/mol were averaged, otherwise deleted.

tDB, 983 variants in 42 proteins. Further filtered to contain only X-ray structures without ligands.

STRUM [20]

Q3421 Single AASs, protein structure available. Weighted averaging of values when several experiments. Measurements close to pH 7 and temperature close to 25°C, without additives, were favoured.

Q306 variants in 32 proteins, sequence identity <60% to S2648 [8].

Meta predictor [21]

605 Single AASs in 60 proteins, manually checked. Measurements at pH 5-9 and temperature 20-30°C. Proteins with cofactors or prosthetic groups deleted. Excluded cases used to train the constituent method. Corrected for the sign of ΔΔG values and energy units.

There are additional papers with ProTherm selections, but since they have just used subsets listed above they are not mentioned in here.

Supplementary Table S1. Distribution of amino acid substitutions in the dataset. The original amino acids are in rows and variant residues in columns.

	A	C	D	E	F	G	H	I	K	L	M	N	P	Q	R	S	T	V	W	Y	Total
A	0	2	3	3	1	18	2	2	3	6	4	1	8	2	2	9	7	8	1	1	83
C	7	0	0	0	0	1	0	2	0	1	1	0	0	0	0	11	3	6	0	0	32
D	32	2	0	9	3	8	9	3	14	3	1	25	3	4	3	6	4	2	2	2	135
E	25	2	5	0	3	5	2	1	27	4	3	5	2	24	2	2	3	6	1	2	124
F	18	1	0	0	0	2	1	1	1	9	3	0	0	0	0	1	1	4	4	8	54
G	21	1	3	3	1	0	1	0	1	1	0	2	3	2	4	6	0	6	0	0	55
H	8	1	2	2	0	4	0	0	1	3	0	2	2	5	2	1	2	0	1	6	42
I	28	3	1	1	4	9	1	0	0	7	9	1	0	0	0	2	10	29	2	1	108
K	29	0	3	22	2	8	3	1	0	0	5	5	2	17	8	2	0	4	1	1	113
L	43	4	1	2	6	6	2	6	1	0	13	1	2	1	5	0	4	14	2	2	115
M	9	1	1	1	2	2	0	6	2	6	0	0	0	0	0	0	2	4	0	1	37
N	17	0	16	1	1	2	3	3	4	1	2	0	0	1	0	5	1	1	0	0	58
P	30	0	0	0	0	6	0	0	0	2	0	1	0	0	1	5	0	1	1	1	48
Q	13	1	0	3	0	4	1	1	2	2	1	1	2	0	2	1	0	0	0	2	36
R	18	3	1	8	1	5	7	1	9	2	3	1	1	7	0	5	1	1	1	1	76
S	23	2	5	2	2	8	2	1	2	0	0	1	1	1	0	0	5	3	0	1	59
T	27	4	7	4	1	10	2	7	1	2	1	5	2	4	3	16	0	23	1	1	121
V	48	11	0	2	4	18	3	23	3	12	9	1	3	1	1	6	26	0	0	4	175
W	2	0	0	0	9	0	2	0	0	1	0	0	0	0	0	0	0	0	0	4	18
Y	12	2	2	0	35	4	2	0	1	3	0	2	1	1	1	2	0	1	6	0	75
Total	410	40	50	63	75	120	43	58	72	65	55	54	32	70	34	80	69	113	23	38	1564

Supplementary Table S2. Distribution of the cleaned dataset to subsets for method training and testing.

Datasets	Variations	Proteins (families)	Increase	Decrease	No-Change
train1 [a]	280	27(13)	44	156	80
train2	279	23(19)	43	151	85
train3	280	10(7)	41	156	83
train4	279	17(12)	41	142	96
train5	281	12(7)	41	167	73
Blind-test set	165	10(10)	23	92	50
total	1564	99(67)	233	864	467

[a] Parts 1-5 denote to the 5 partitions used in 5-fold cross validation.

Supplementary Table S3. Features used to train PON-tstab.

Feature name	Description	Importance factor in RF	Reference
**Layer1： Features selected for the decrease/no decrease classifier**			
pssm_wild	Position specific score for the wild type residue	76.4	
BULH740101	Transfer free energy to surface	63.6	[22]
ZHOH040101	The stability scale from the knowledge-based atom-atom potential	77.5	[23]
pssm_mut	Position specific score for the variant residue	53.4	
ZHAC000102	Environment-dependent residue contact energies	68.5	[24]
T	Temperature	66.3	
NonPolarAA	Frequency of non-polar amino acids in a 23 amino acid neighbor window	58.1	
BAEK050101	Prediction of protein inter-domain linker regions by a hidden Markov model	67.7	[25]
**Layer2: Features selected for the increase/no effect classifier**			
T	Temperature	61.7	
NADH010105	Hydropathy scale based on self-information values in the two-state model (25% accessibility)	56.7	[26]
ZIMJ680102	Bulkiness	61.2	[27]

Supplementary Table S4. Sequence-based features from ProtDCal.

**Feature**	**Description**
Gw(U)	Free energy contribution from the entropy of the first shell of water molecules in an unfolded state
Gs(U)	Interfacial free energy contribution of an unfolded state
W(U)	Number of water molecules close to a residue in an unfolded state
Mw	Molecular weight
HP	Kyte-Doolitle's hydrophobicity scale
IP	Isoelectric point
ECI	Electronic charge index
Vm	Amino acid volume
Anp	Nonpolar area
Z1	Composed parameter related with hydrophilicity
Z2	Composed parameter related with steric features
Z3	Composed parameter related with electronic features
ISA	Isotropic surface area
At	Estimated solvent accessible surface area of residues in fully exposed states
Ap	Polar area
Pa	Levitt's Probability of adopting alpha helix conformation
Pb	Levitt's Probability of adopting beta sheet conformation
Pt	Levitt's Probability of adopting beta turn conformation
TAE energy	Transferable Atom Equivalent energy

References

1. Capriotti, E.; Fariselli, P.; Casadio, R. A neural-network-based method for predicting protein stability changes upon single point mutations. *Bioinformatics* **2004,** *20 Suppl 1*,i63-8,10.1093/bioinformatics/bth928.
2. Capriotti, E.; Fariselli, P.; Casadio, R. I-Mutant2.0: predicting stability changes upon mutation from the protein sequence or structure. *Nucleic Acids Res* **2005,** *33*,W306-10,10.1093/nar/gki375.
3. Cheng, J.; Randall, A.; Baldi, P. Prediction of protein stability changes for single-site mutations using support vector machines. *Proteins* **2006,** *62*,1125-32,10.1002/prot.20810.
4. Saraboji, K.; Gromiha, M. M.; Ponnuswamy, M. N. Average assignment method for predicting the stability of protein mutants. *Biopolymers* **2006,** *82*,80-92,10.1002/bip.20462.
5. Huang, L. T.; Gromiha, M. M.; Ho, S. Y. iPTREE-STAB: interpretable decision tree based method for predicting protein stability changes upon mutations. *Bioinformatics* **2007,** *23*, 1292–3, 10.1093/bioinformatics/btm100.

6. Capriotti, E.; Fariselli, P.; Rossi, I.; Casadio, R. A three-state prediction of single point mutations on protein stability changes. *BMC Bioinformatics* **2008,** *9 Suppl 2*,S6,10.1186/1471-2105-9-s2-s6.
7. Masso, M.; Vaisman, II. Accurate prediction of stability changes in protein mutants by combining machine learning with structure based computational mutagenesis. *Bioinformatics* **2008,** *24*,2002-9,10.1093/bioinformatics/btn353.
8. Dehouck, Y.; Grosfils, A.; Folch, B.; Gilis, D.; Bogaerts, P.; Rooman, M. Fast and accurate predictions of protein stability changes upon mutations using statistical potentials and neural networks: PoPMuSiC-2.0. *Bioinformatics* **2009,** *25*,2537-43,10.1093/bioinformatics/btp445.
9. Potapov, V.; Cohen, M.; Schreiber, G. Assessing computational methods for predicting protein stability upon mutation: good on average but not in the details. *Protein Eng Des Sel* **2009,** *22*,553-60,10.1093/protein/gzp030.
10. Guerois, R.; Nielsen, J. E.; Serrano, L. Predicting changes in the stability of proteins and protein complexes: a study of more than 1000 mutations. *J Mol Biol* **2002,** *320*,369-87,10.1016/s0022-2836(02)00442-4.
11. Khan, S.; Vihinen, M. Performance of protein stability predictors. *Hum Mutat* **2010,** *31*,675-84,10.1002/humu.21242.
12. Zhang, Z.; Wang, L.; Gao, Y.; Zhang, J.; Zhenirovskyy, M.; Alexov, E. Predicting folding free energy changes upon single point mutations. *Bioinformatics* **2012,** *28*,664-71,10.1093/bioinformatics/bts005.
13. Yang, Y.; Chen, B.; Tan, G.; Vihinen, M.; Shen, B. Structure-based prediction of the effects of a missense variant on protein stability. *Amino Acids* **2013,** *44*,847-55,10.1007/s00726-012-1407-7.
14. Folkman, L.; Stantic, B.; Sattar, A. Feature-based multiple models improve classification of mutation-induced stability changes. *BMC Genomics* **2014,** *15 Suppl 4*,S6,10.1186/1471-2164-15-s4-s6.
15. Folkman, L.; Stantic, B.; Sattar, A.; Zhou, Y. EASE-MM: Sequence-Based Prediction of Mutation-Induced Stability Changes with Feature-Based Multiple Models. *J Mol Biol* **2016,** *428*,1394-1405,10.1016/j.jmb.2016.01.012.
16. Pires, D. E.; Ascher, D. B.; Blundell, T. L. mCSM: predicting the effects of mutations in proteins using graph-based signatures. *Bioinformatics* **2014,** *30*,335-42,10.1093/bioinformatics/btt691.
17. Pucci, F.; Bourgeas, R.; Rooman, M. Predicting protein thermal stability changes upon point mutations using statistical potentials: Introducing HoTMuSiC. *Sci Rep* **2016,** *6*,23257,10.1038/srep23257.
18. Jia, L.; Yarlagadda, R.; Reed, C. C. Structure Based Thermostability Prediction Models for Protein Single Point Mutations with Machine Learning Tools. *PLoS One* **2015,** *10*,e0138022,10.1371/journal.pone.0138022.
19. Getov, I.; Petukh, M.; Alexov, E. SAAFEC: Predicting the Effect of Single Point Mutations on Protein Folding Free Energy Using a Knowledge-Modified MM/PBSA Approach. *Int J Mol Sci* **2016,** *17*,512,10.3390/ijms17040512.
20. Quan, L.; Lv, Q.; Zhang, Y. STRUM: structure-based prediction of protein stability changes upon single-point mutation. *Bioinformatics* **2016,** *32*,2936-46,10.1093/bioinformatics/btw361.
21. Broom, A.; Jacobi, Z.; Trainor, K.; Meiering, E. M. Computational tools help improve protein stability but with a solubility tradeoff. *J Biol Chem* **2017,** *292*,14349-14361,10.1074/jbc.M117.784165.
22. Bull, H. B.; Breese, K. Surface tension of amino acid solutions: a hydrophobicity scale of the amino acid residues. *Arch Biochem Biophys* **1974,** *161*,665-70.
23. Zhou, H.; Zhou, Y. Quantifying the effect of burial of amino acid residues on protein stability. *Proteins* **2004,** *54*,315-22,10.1002/prot.10584.
24. Zhang, C.; Kim, S. H. Environment-dependent residue contact energies for proteins. *Proc Natl Acad Sci U S A* **2000,** *97*,2550-5,10.1073/pnas.040573597.
25. Bae, K.; Mallick, B. K.; Elsik, C. G. Prediction of protein interdomain linker regions by a hidden Markov model. *Bioinformatics* **2005,** *21*,2264-70,10.1093/bioinformatics/bti363.
26. Naderi-Manesh, H.; Sadeghi, M.; Arab, S.; Moosavi Movahedi, A. A. Prediction of protein surface accessibility with information theory. *Proteins* **2001,** *42*,452-9.
27. Zimmerman, J. M.; Eliezer, N.; Simha, R. The characterization of amino acid sequences in proteins by statistical methods. *J Theor Biol* **1968,** *21*,170-201.
